# High mesothelin expression is correlated with non-squamous cell histology and poor survival in cervical cancer: a retrospective study

**DOI:** 10.1186/s12885-022-10277-0

**Published:** 2022-11-24

**Authors:** Shigemasa Takamizawa, Shu Yazaki, Yuki Kojima, Hiroshi Yoshida, Rui Kitadai, Tadaaki Nishikawa, Tatsunori Shimoi, Kazuki Sudo, Hitomi Sumiyoshi Okuma, Maki Tanioka, Emi Noguchi, Masaya Uno, Mitsuya Ishikawa, Tomoyasu Kato, Yasuhiro Fujiwara, Kan Yonemori

**Affiliations:** 1 Department of Medical Oncology, National Cancer Center Hospital, 5-1-1 Tsukiji, Chuo-Ku, Tokyo, 104-0045 Japan; 2 Department of Diagnostic Pathology, National Cancer Center Hospital, Tokyo, Japan; 3 Department of Gynecology, National Cancer Center Hospital, Tokyo, Japan

**Keywords:** Cervical cancer, Mesothelin, Squamous cell carcinoma, Targeted therapy

## Abstract

**Background:**

Mesothelin (MSLN) is a cell-surface glycoprotein found in various solid tumours. Cancer therapies targeting MSLN have been developed in recent years; however, the available information on MSLN expression in cervical cancer is limited. This study aimed to evaluate MSLN expression in various histological types of cervical cancer and examine its relationship with prognosis.

**Methods:**

This retrospective study included patients with cervical cancer who underwent primary surgery between January 2000 and December 2020 at our institution. MSLN expression was evaluated by immunohistochemistry using clone SP74 and defined as positive if MSLN was expressed at any intensity. High MSLN expression was defined as an intensity of ≥ 2 + in ≥ 30% of tumour cells. The association between MSLN expression and clinicopathological factors was evaluated.

**Results:**

Overall, 123 patients were identified, and 140 tumour samples, including 17 paired primary and metastatic samples, were evaluated. Concerning histological type, 67 patients had squamous cell carcinoma (SCC), whereas 56 had non-SCC. MSLN expression was observed in 98.4% (121/123) of primary tumours. High MSLN expression was observed in 63.4% of samples (78/123), but it differed between the histological types (49.2% for SCC vs. 80.4% for non-SCC, *p* < 0.001). There was a significant correlation between MSLN expression in primary and metastatic lesions (Rs = 0.557, *p* = 0.015). In patients with common histological types, overall survival (OS) was shorter in the high MSLN expression group than in the low MSLN expression group (hazard ratio, 3.53; 95% confidence interval, 1.16–15.3, *p* = 0.03).

**Conclusions:**

MSLN was highly expressed in patients with cervical cancer, especially in those with non-SCC. High MSLN expression in the primary lesion was significantly associated with poor OS, and its expression was maintained in metastatic lesions. Our findings indicate that MSLN may be an attractive therapeutic target for cervical cancer.

**Trial registration:**

Retrospectively registered. 2014-393. 1 June 2015

**Supplementary Information:**

The online version contains supplementary material available at 10.1186/s12885-022-10277-0.

## Background

Cervical cancer is the fourth most common cancer among women globally [1], and there has been an increasing trend in its incidence, especially in young women [2, 3]. Although there has been a decrease in the incidence of cervical cancer in Western countries, due to vaccination against the high-risk human papillomavirus, its incidence in Asia is still high [2, 4]. The prognosis of metastatic or recurrent cervical cancer is poor with a median survival of only 17 months [5, 6]. After patients become refractory to first-line platinum-based chemotherapy, second-line and subsequent chemotherapeutic strategies have limited efficacy [7]. Therefore, it is crucial to investigate novel treatment strategies to improve prognosis in patients with cervical cancer.

Mesothelin (MSLN) is a cell-surface glycoprotein, which is highly expressed in many cancers, including malignant mesothelioma, pancreatic cancer, and ovarian cancer [8]. Aberrant MSLN expression is thought to play a significant role in promoting proliferation, cell migration, and invasion [9]. Cancer therapies targeting MSLN have been developed in recent years. Chimeric anti-MSLN antibodies, anti-MSLN immunotoxins, antibody–drug conjugates, chimeric antigen receptor T-cell therapies, and *Listeria monocytogenes*-expressing MSLN are under clinical evaluation [8]. Hence, MSLN has been found to be an attractive target for use in cancer treatment.

Few studies have evaluated MSLN expression in patients with cervical cancer. A previous report showed that 42.4% of 125 patients with squamous cell carcinoma (SCC) of the cervix expressed MSLN using anti-MSLN antibodies (MSVA-235) [10]. Another study that used anti-MSLN antibodies (clone 5B2) found that approximately 60% of patients with cervical cancer express MSLN, with its expression being lower in SCC than in adenocarcinoma (AC) [11]. However, staining of clone 5B2 is different from that of clone SP74 [12], which has been used in MSLN-targeted clinical trials for other carcinomas [13]. Moreover, the prognostic value of MSLN expression, its expression profiles in rare and aggressive histological types, such as gastric-type adenocarcinoma (GAS) and neuroendocrine carcinoma (NEC), and differences in its expression levels between primary and metastatic tumours remain unclear. Thus, we aimed to evaluate MSLN expression in cervical cancer of various histological types using anti-MSLN antibodies (clone SP74) and to further examine the prognosis and changes in its expression in paired metastatic tumours.

## Methods

### Study cohort

We identified patients with cervical cancer who underwent surgery as the primary treatment for cancer at the National Cancer Center Hospital (Tokyo, Japan) between January 2000 and December 2020. In addition, among these patients, we identified those who subsequently developed metastatic disease and underwent tumour biopsy for paired metastatic tumours. Patients lacking sufficient primary tumour tissues were excluded from this study. We retrospectively collected clinical and pathological data, such as information on age, histology, and clinical stage, as defined by the International Federation of Gynecology and Obstetrics (FIGO) in 2008 [14], lymph node metastasis, adjuvant treatment, and survival time after surgery.

This study was approved by the Institutional Review Board of the National Cancer Center (Tokyo, Japan) (No. 2014–393). The requirement to obtain informed consent was waived due to the retrospective nature of the study. The study was conducted in accordance with the principles of the Declaration of Helsinki.

### Pathological diagnoses

Pathological diagnoses were confirmed by at least two gynaecological pathologists. Given that the study period was approximately 20 years, the permanent slides of all the cases were microscopically reviewed, and final diagnoses were confirmed based on the 2014 World Health Organization classification of cervical cancer [15]. In this study, we separated usual-type endocervical AC and GAS for subsequent analyses because these two types of ACs have been reported to have significantly distinct aetiologies and clinicopathological features [16, 17].

### Immunohistochemical staining and evaluation

Haematoxylin and eosin-stained slides for each case were reviewed to obtain representative sections. New 4 μm-thick whole sections were prepared from formalin-fixed paraffin-embedded surgical specimens and immunohistochemically stained. We evaluated MSLN expression using the VENTANA MSLN (SP74) IHC assay (clone SP74, rabbit monoclonal antibody, ready to use, EDTA buffer, Roche Diagnostics, Rotkreuz, Switzerland) on the Ventana BenchMark XT automated immunostainer (Roche Diagnostics) according to the manufacturer’s instructions.

Membrane MSLN staining was performed by a trained pathologist. The staining intensity for evaluating MSLN protein expression was recorded using a four-tiered scoring system (0: no detectable signal, 1 + : weak, 2 + : moderate, and 3 + : strong), which is described in the antibody kit's instructions and previous reports using the same clone of the anti-MSLN antibody [18–20]. In addition, we used a quantitative analysis of IHC (H-Score) for a more detailed evaluation of the expression status. The H-Score was obtained using the following formula: 3 × percentage of strongly stained (3 +) cells + 2 × percentage of moderately stained (2 +) cells + percentage of weakly stained (1 +) tumour cells. We stained one representative section of each tumour and evaluated these at low magnification (× 40) to high magnification (× 200). The maximum and minimum H-scores were 300 (strong staining for all tumour cells) and 0 (no tumour cell staining), respectively. MSLN positivity was defined as MSLN expression at any intensity [13]. High MSLN expression was defined as an intensity ≥ 2 + in ≥ 30% of tumour cells, based on the findings of a previous study [13].

We used an optical microscope (BX53, Olympus, Tokyo, Japan) connected to a digital camera (DP21, Olympus, Tokyo, Japan) to acquire histopathological images according to the manufacturer’s instructions.

### Statistical analysis

We evaluated differences in MSLN expression with respect to clinicopathological factors and primary and metastatic lesions. Continuous variables were reported as the median (range and interquartile range) and compared using the Mann–Whitney U test. Categorical variables were reported as numbers and percentages and compared using the chi-squared test. Spearman's rank correlation coefficient was used to compare the H-scores between primary and metastatic tumours. Relapse-free survival (RFS) was defined as the time from surgery to the first relapse or death from any cause. Overall survival (OS) was defined as the time from surgery until death due to any cause. RFS and OS were analysed using the Kaplan–Meier method. The log-rank test was used to compare survival between the groups. Univariate and multivariate cox regression analyses were performed to determine the prognostic impact of MSLN expression. All tests were two-tailed, and the significance level was set at α = 0.05. Statistical analyses were performed using STATA (version 15.1; StataCorp, College Station, TX, USA), GraphPad Prism version 8.0 (GraphPad Software, San Diego, California, USA), and JMP 14.3.0 for Windows statistical software (SAS Institute Japan Inc., Cary, NC, USA).

## Results

### Patient characteristics

A total of 123 patients were included in the analysis. Among them, 17 had paired metachronous metastatic tumours for which MSLN expression could be assessed. Patient characteristics are presented in Table 1. The median age was 45 years (range, 26–72 years). There were 67 (54.5%), 27 (22.0%), 13 (10.6%), 12 (9.8%), and 4 (3.3%) patients with SCC, AC, adenosquamous carcinoma (ASC), GAS, and NEC, respectively. Ninety-six patients (78%) had FIGO stage I disease, while 27 (22%) had FIGO stage II disease. Lymph node metastasis was observed in 45 patients (36.6%). For postoperative treatment, 44 patients (35.8%) received postoperative radiotherapy, whereas 13 (10.6%) received postoperative chemoradiotherapy.

**Table 1:** Primary tumour characteristics of 123 patients

		MSLN-low (*n* = 45)	MSLN-high (*n* = 78)	Total (*n* = 123)	*p*-value
Age (years), median (range)		47 (28–66)	44 (26–72)	45 (26–72)	0.54
Histology, n (%)	SCC	34 (75.6)	33 (42.3)	67 (54.5)	** < 0.001**
	AC	4 (8.9)	23 (29.5)	27 (22)	
	ASC	2 (4.4)	11 (14.1)	13 (10.6)	
	GAS	1 (2.2)	11 (14.1)	12 (9.8)	
	NEC	4 (8.9)	0 (0)	4 (3.3)	
FIGO stage (2008), n (%)	I	34 (75.6)	62 (79.5)	96 (78.0)	0.61
	II	11 (24.4)	16 (20.5)	27 (22.0)	
Tumour size, n (%)	≤ 4 cm	27 (60)	40 (51.3)	67 (54.5)	0.35
	> 4 cm	18 (40)	38 (48.7)	56 (45.5)	
Lymph node metastasis, n (%)	Yes	14 (31.1)	31 (39.7)	45 (36.6)	0.34
	No	31 (68.9)	47 (60.3)	78 (63.4)	
Lymphovascular space invasion, n (%)	Yes	20 (44.4)	53 (67.9)	73 (59.3)	**0.01**
	No	25 (55.6)	25 (32.1)	50 (40.7)	
Postoperative RT, n (%)	Yes	12 (26.7)	32 (41)	44 (35.8)	0.11
	No	33 (73.3)	46 (59)	79 (64.2)	
Postoperative CRT, n (%)	Yes	2 (4.4)	11 (14.1)	13 (10.6)	0.09
	No	43 (95.6)	67 (85.9)	110 (89.4)	

*AC* Adenocarcinoma, *ASC* Adenosquamous carcinoma, *CRT* Chemoradiotherapy, *FIGO* International Federation of Gynecology and Obstetrics, *GAS* Gastric-type adenocarcinoma, *MSLN* Mesothelin; n: number, *NEC* Neuroendocrine carcinoma, *RT* Radiotherapy, *SCC* Squamous cell carcinoma

### MSLN expression and differences in histological types

**Fig. 1:**
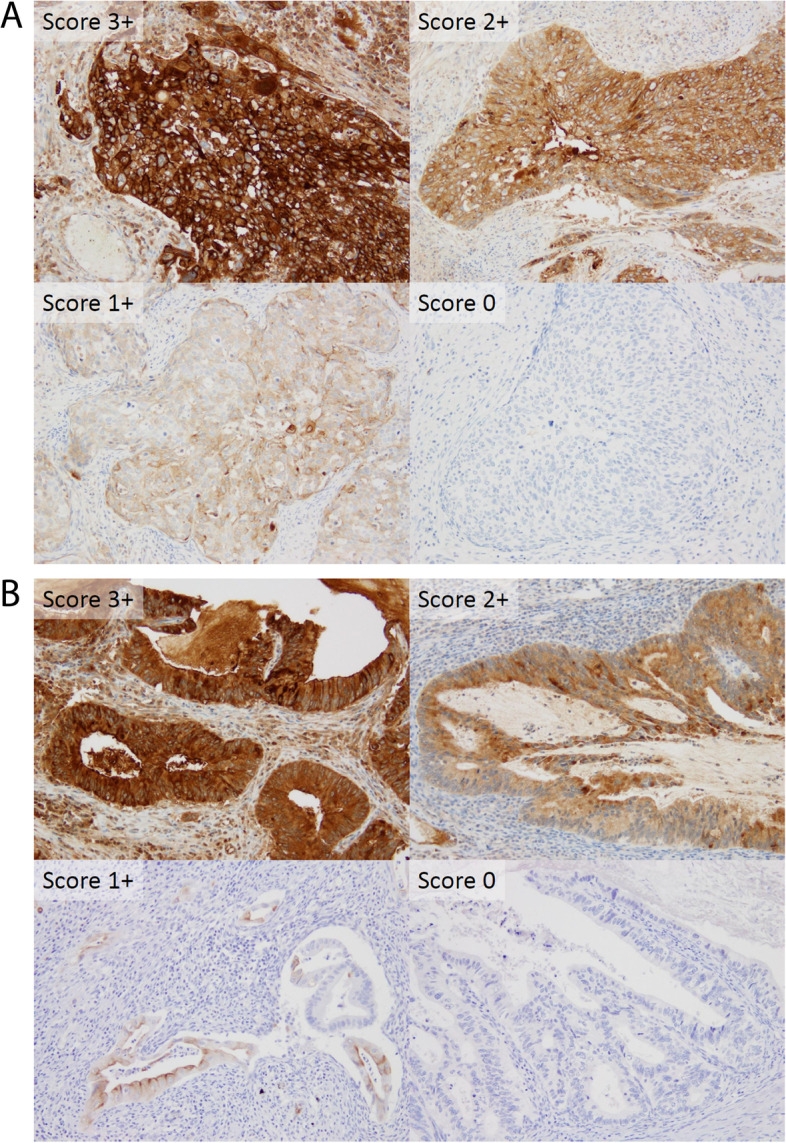
Staining intensity patterns for mesothelin expression. Representative microphotographs of 0, 1 + , 2 + , and 3 + staining intensity patterns for mesothelin expression in (**A**) squamous cell carcinoma and (**B**) adenocarcinoma (original magnification × 200)

MSLN expression was observed in 98.4% (121/123) of the patients. High MSLN expression was observed in 63.4% (78/123) of primary tumour samples. Figure 1A and B show staining intensity patterns for MSLN expression in SCC and AC, respectively. Supplementary Fig. 1 shows the representative micrographs of MSLN expression in each histological type.

**Fig. 2:**
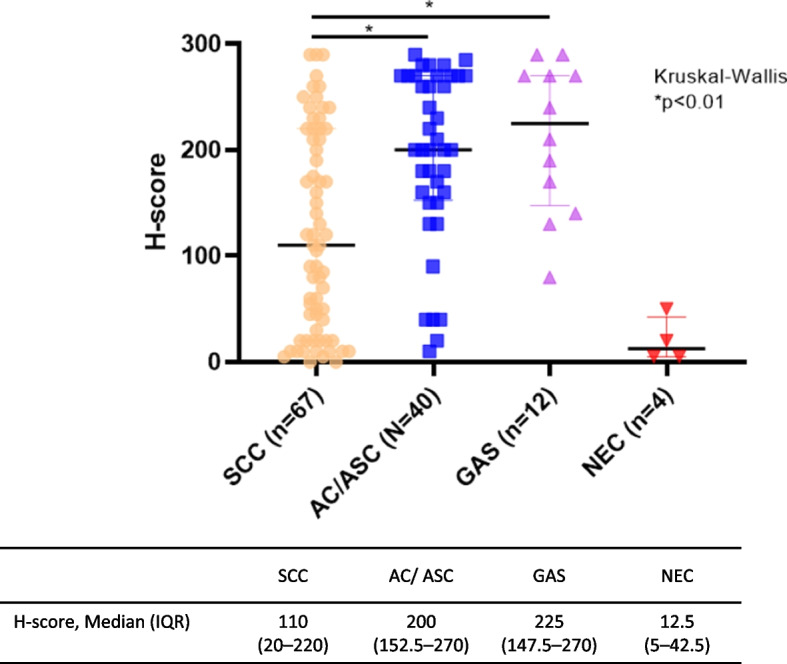
MSLN expression with respect to histology. H-scores for MSLN expression in the different histological types were compared. The dots represent H-score in each patient; the horizontal line represents the median; the whiskers represent interquartile range; the orange, blue, pink, and red dots represent SCC, AC/ASC, GAS, and NEC, respectively. AC: adenocarcinoma; ASC: adenosquamous carcinoma; GAS: gastric-type adenocarcinoma; MSLN: mesothelin; NEC: neuroendocrine carcinoma; SCC: squamous cell carcinoma

High MSLN expression was not associated with age, tumour size, FIGO stage, or lymph node metastasis; however, it was associated with histological type and lymphovascular space invasion. High MSLN expression was more frequent in non-SCC than in SCC (80.4% [45/56] in non-SCC vs. 49.2% [33/67] in SCC, *p* < 0.001) (Table 1). Figure 2 shows H-score distribution with respect to histology. The median H-scores for AC/ASC and GAS were significantly higher than those for SCC (200 [152.5–270] for AC/ASC vs. 110 [20–220] for SCC, *p* < 0.01; 225 [147.5–270] for GAS vs. 110 [20–220] for SCC, *p* < 0.01).

### MSLN expression in primary and metastatic tumours

**Fig. 3:**
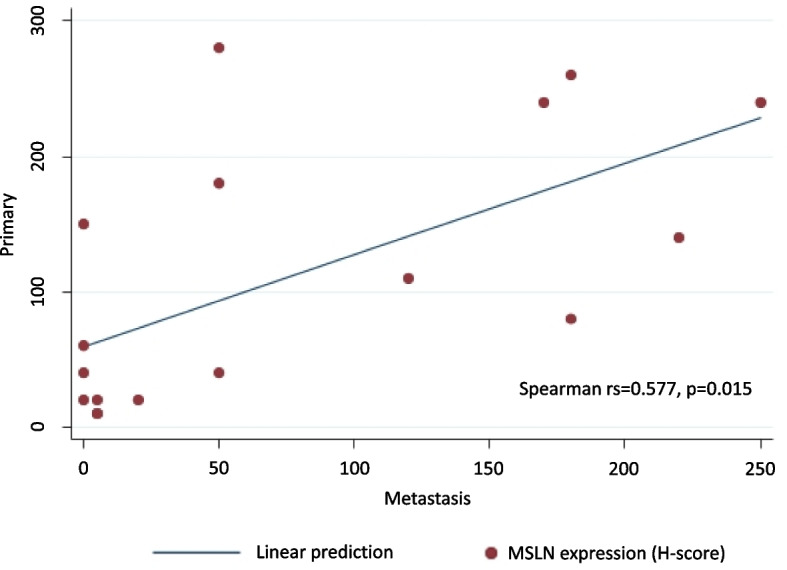
Correlation between mesothelin expression in primary and metastatic lesions. Correlation between MSLN expression in primary and metastatic lesions. The red dots represent the H-score for each patient. MSLN: mesothelin

Analysis of the paired primary and metastatic tumours of 17 patients who had paired metachronous metastatic tumours showed a significant correlation between MSLN expression in primary and metastatic lesions (Spearman’s rank correlation coefficient; Rs = 0.557, *p* = 0.015, Fig. 3). Four of these patients (23.5%) showed a discordance in MSLN expression between primary and metastatic tumours. Three patients with high MSLN expression in their primary tumours showed low MSLN expression in their metastatic tumours. One patient showed low MSLN expression in the primary tumour and high MSLN expression in the metastatic tumour. Supplementary Fig. 2 shows a representative case in which MSLN expression changed between primary and metastatic tumours.

### Association between MSLN expression and survival

We evaluated the association between MSLN expression in primary tumours and survival. The median follow-up period was 68.9 months (95% confidence interval [CI], 63.3–76.2). The median RFS and OS were not significantly different between the high and low MSLN expression groups in all populations (RFS: hazard ratio [HR], 1.26; 95% CI, 0.68–2.43; *p* = 0.46; OS: HR, 2.39; 95% CI, 0.95–7.27; *p* = 0.08) (Supplementary Fig. 3A and B).

**Fig. 4:**
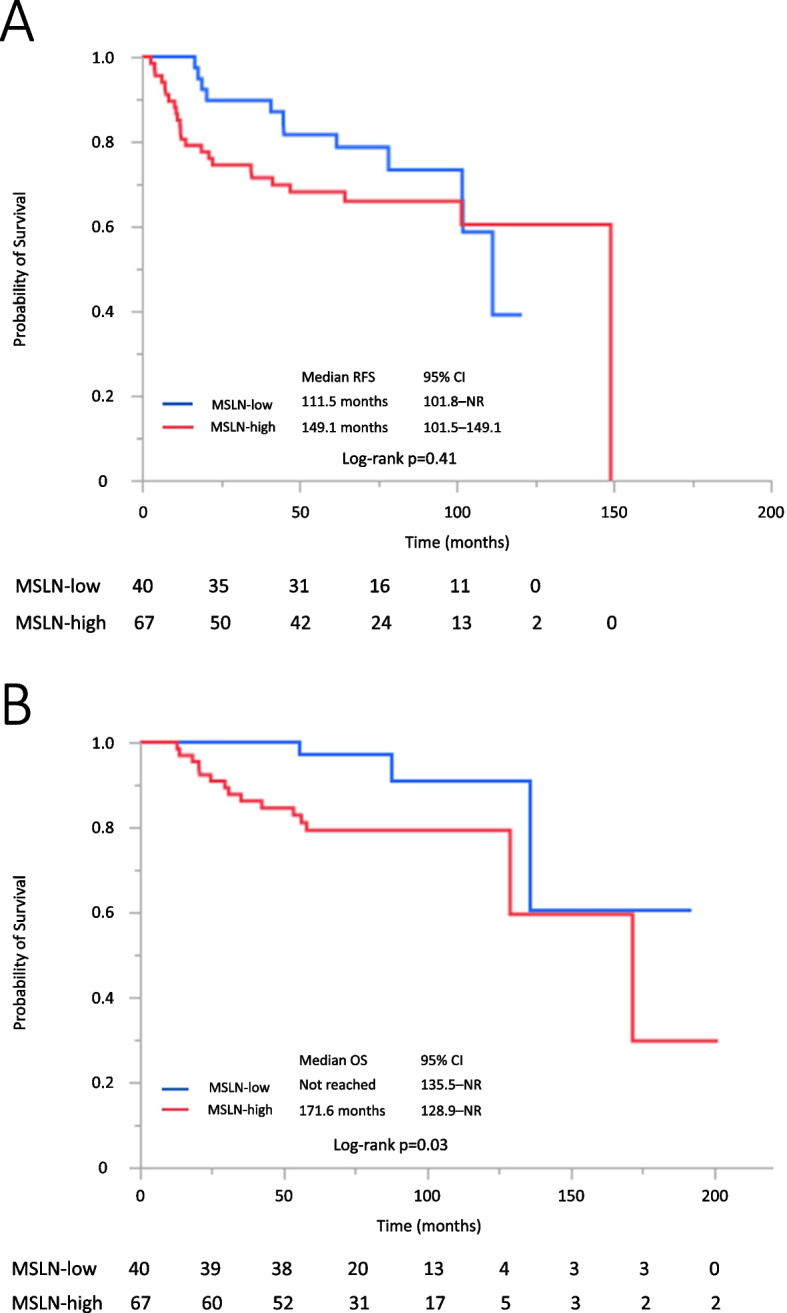
Kaplan–Meier RFS and OS analysis in patients with SCC, AC, and ASC. Kaplan–Meier RFS and OS analysis with respect to MSLN expression (**A**) RFS and (**B**) OS in patients with SCC, AC, and ASC with high and low MSLN expression. AC: adenocarcinoma; ASC: adenosquamous carcinoma; MSLN: mesothelin; OS: overall survival; RFS: relapse-free survival; SCC: squamous cell carcinoma

As GAS and NEC are associated with a significantly poorer prognosis, we subsequently evaluated the association between MSLN expression and survival in 107 patients with common histological types, i.e., SCC, AC, and ASC. RFS tended to be shorter in the high MSLN expression group than in the low MSLN expression group (HR, 1.34; 95% CI, 0.68–2.79; *p* = 0.41) (Fig. 4A). OS was shorter in the high MSLN expression group than in the low MSLN expression group (HR, 3.53; 95% CI, 1.16–15.3; *p* = 0.03) (Fig. 4B). In 67 patients with SCC, the median RFS and OS tended to be shorter in the high MSLN expression group than in the low MSLN expression group (RFS: HR, 1.96; 95% CI, 0.79–5.27; *p* = 0.15; OS: HR, 3.82; 95% CI, 0.94–25.49; *p* = 0.06) (Supplementary Fig. 4A and B). In 40 patients with AC and ASC, the median RFS tended to be longer in the high MSLN expression group than in the low MSLN expression group (HR, 0.54; 95% CI, 0.19–1.75; *p* = 0.29) (Supplementary Fig. 4C). The median OS tended to be shorter in the high MSLN expression group than in the low MSLN expression group (HR, 1.53; 95% CI, 0.25–29.3; *p* = 0.69) (Supplementary Fig. 4D).

In the multivariate analyses adjusting for age, histology, FIGO stage, lymph node metastasis, and presence of post-operative radiotherapy and chemoradiotherapy, MSLN expression was significantly associated with OS (HR, 3.70; 95% CI, 1.09–17.6; *p* = 0.034), but not RFS (HR, 0.98; 95% CI, 0.47–2.15; *p* = 0.96) (Supplementary Table 1).

## Discussion

To the best of our knowledge, this is the largest study to evaluate MSLN expression in various histological types and examine prognostic outcomes in cervical cancer. MSLN expression is known to be positive in pancreatic adenocarcinoma (82%), mesothelioma (85%), and ovarian cancer (70%) [8]. In our study, MSLN expression was observed in 98.4% of patients with cervical cancer, with MSLN being highly expressed in 63.4% of them. High MSLN expression was more frequent in non-SCC than in SCC.

MSLN expression differed with respect to histological type. High MSLN expression was significantly more frequently observed in non-SCC than in SCC (80.4% vs. 49.2%). These results are consistent with those of a previous study in which MSLN expression was found to be high in AC and weak or modest in SCC in non-small cell lung cancer [21]. Jöhrens et al. analysed MSLN expression in 79 patients with cervical cancer (26 had AC and 53 had SCC) and found that it was positive in 57% of patients with SCC, which was lower than in patients with AC, with a positivity rate of 77% [11]. Although these results are similar to those obtained in the present study, direct comparison is difficult as they used different anti-MSLN antibody clones (clone 5B2 vs. clone SP74), staining evaluation methods, and histological types [11]. To the best of our knowledge, our study is the first to evaluate MSLN expression in cervical cancer using the anti-MSLN antibodies (clone SP74) previously used in MSLN-targeted clinical trials [13]. Patients with cervical AC or GAS are more resistant to standard treatments and have a poorer prognosis than those with SCC [22, 23]. Thus, MSLN may become an attractive therapeutic target for patients with cervical AC or GAS.

High MSLN expression was associated with poor OS in patients with common histological cervical cancer types. Previous studies also demonstrated that high MSLN expression was correlated with poor prognosis in other cancer types, including ovarian cancer [24], biliary cancer [25, 26], pancreatic AC [27, 28], lung AC [29, 30], and triple-negative breast cancer [31, 32]. Cheng et al. found that MSLN expression was higher in patients with platinum-resistant ovarian cancer than in those with platinum-sensitive ovarian cancer [33]. Thus, MSLN may play a role in chemoresistance and tumour progression in cervical cancer.

A correlation has been observed between MSLN expression in primary and metastatic lesions in cervical cancer. A previous study showed a positive correlation between MSLN expression in primary and metastatic tumours in patients with colorectal cancer [34]. Furthermore, the concordance rate of high MSLN expression in primary and metastatic tumours was over 75% in our study. While MSLN-targeted therapy is being developed for metastatic and recurrent cancers, this high concordance rate may allow us to use archived specimens of primary tumours for screening even in patients with metastatic cancer. However, three patients showing high MSLN expression in primary lesions had low expression in metastatic lesions; these patients may not respond to MSLN-targeted therapy. Further studies are needed to determine the factors that may influence MSLN expression in metastases.

Several MSLN-targeted therapies have demonstrated clinical activity in early phase clinical trials. In a phase I/II study on the efficacy of the MSLN-targeting recombinant immunotoxin, LMB-100, in combination with nab-paclitaxel for the treatment of advanced pancreatic cancer, a correlation between CA19-9 response to investigational treatment and MSLN expression was suggested [35, 36]. The MSLN-directed antibody–drug conjugate, anetumab ravtansine, which consists of a human anti-MSLN antibody conjugated to the maytansinoid tubulin inhibitor, DM4, via a disulfide-containing linker, was found to be active against malignant mesothelioma and ovarian cancer with high MSLN expression [13]. These MSLN-targeted therapies may improve treatment outcomes for advanced stage tumours with high MSLN expression.

This study, however, has several limitations. First, it had a retrospective design and a relatively small sample size, with all data obtained from a single institution. Although these are rare populations, the number of patients with uncommon histology and whose MSLN expression was evaluated in both the primary and metastatic site was quite small. Second, the enrolment period of the patients was long; treatment strategies have changed over the years, and this may have affected prognosis. These limitations make it difficult to draw definitive conclusions.

## Conclusions

Over 60% of patients with cervical cancer exhibited high MSLN expression levels. High MSLN expression was substantially more frequent in non-SCC than in SCC and was associated with poor prognosis in patients with cervical cancer of common histological types. There was a significant correlation between MSLN expression in primary and metastatic tumours. Collectively, these findings may motivate further investigation of MSLN-targeted therapy for patients with cervical cancer.

## Supplementary Information

**Additional file 1: Supplementary Figure 1. **Representative microphotographs of mesothelin expression in each histological type. Squamous cell carcinoma (A and B), adenosquamous carcinoma (C and D), endocervical adenocarcinoma (usual-type) (E and F), gastric-type adenocarcinoma (G and H), and small cell neuroendocrine carcinoma (I and J). Haematoxylin & eosin stain: A, C, E, G, and I; immunohistochemical mesothelin staining: B, D, F, H, and J, original magnification ×200. **Supplementary Figure 2.** MSLN expression in paired specimens. Case 1 showed moderate MSLN expression in surgically resected tumour tissue (A, H-score = 120) and similar staining to the biopsy specimen of a metachronous recurrent tumour (B, H-score = 110). In contrast, Case 2 exhibited moderate to high MSLN expression in a surgically resected tumour (C, H-score = 150); however, MSLN expression was not detected in the biopsy specimen of a metastatic tumour (D, H-score = 0) (original magnification ×200). MSLN: mesothelin. **Supplementary Figure 3.** Kaplan–Meier RFS and OS analysis in all patients with respect to MSLN expression. Kaplan–Meier RFS and OS analysis with respect to MSLN expression. (A) RFS and (B) OS in patients with high MSLN expression versus those in patients with low MSLN expression. MSLN: mesothelin; OS: overall survival; RFS: relapse-free survival. **Supplementary Figure 4.** Kaplan–Meier RFS and OS analysis in patients with SCC or patients with AC and ASC with respect to MSLN expression. Kaplan–Meier RFS and OS analysis with respect to MSLN expression. (A) RFS and (B) OS in SCC patients with high MSLN expression versus those in patients with low MSLN expression. (C) RFS and (D) OS in AC and ASC patients with high MSLN expression versus those in patients with low MSLN expression. AC: adenocarcinoma; ASC: adenosquamous carcinoma; MSLN: mesothelin; OS: overall survival; RFS: relapse-free survival; SCC: squamous cell carcinoma.

**Additional file 2:** **Supplementary Table 1A.** Multivariate analyses of relapse-free survival in 107 patients with common histological types. **Supplementary Table 1B.** Multivariate analyses of overall survival in 107 patients with common histological types.

## Data Availability

The datasets generated during and/or analysed during the current study are available from the corresponding author on reasonable request.

## Abbreviations

AC: Adenocarcinoma
ASC: Adenosquamous carcinoma
CI: Confidence interval
FIGO: International Federation of Gynecology and Obstetrics
GAS: Gastric-type adenocarcinoma
HR: Hazard ratio
MSLN: Mesothelin
NEC: Neuroendocrine carcinoma
OS: Overall survival
RFS: Relapse-free survival
SCC: Squamous cell carcinoma
