# Supplementary material for: High mesothelin expression is correlated with non-squamous cell histology and poor survival in cervical cancer: a retrospective study
Source: BMC Cancer. 2022 Nov 24;22:1215. doi: 10.1186/s12885-022-10277-0 (PMC9701073; doi:10.1186/s12885-022-10277-0)
Supplement: Supplementary file 1 — Additional file 1: Supplementary Figure 1. Representative microphotographs of mesothelin expression in each histological type. Squamous cell carcinoma (A and B), adenosquamous carcinoma (C and D), endocervical adenocarcinoma (usual-type) (E and F), gastric-type adenocarcinoma (G and H), and small cell neuroendocrine carcinoma (I and J). Haematoxylin & eosin stain: A, C, E, G, and I; immunohistochemical mesothelin staining: B, D, F, H, and J, original magnification ×200. Supplementary Figure 2. MSLN expression in paired specimens. Case 1 showed moderate MSLN expression in surgically resected tumour tissue (A, H-score = 120) and similar staining to the biopsy specimen of a metachronous recurrent tumour (B, H-score = 110). In contrast, Case 2 exhibited moderate to high MSLN expression in a surgically resected tumour (C, H-score = 150); however, MSLN expression was not detected in the biopsy specimen of a metastatic tumour (D, H-score = 0) (original magnification ×200). MSLN: mesothelin. Supplementary Figure 3. Kaplan–Meier RFS and OS analysis in all patients with respect to MSLN expression. Kaplan–Meier RFS and OS analysis with respect to MSLN expression. (A) RFS and (B) OS in patients with high MSLN expression versus those in patients with low MSLN expression. MSLN: mesothelin; OS: overall survival; RFS: relapse-free survival. Supplementary Figure 4. Kaplan–Meier RFS and OS analysis in patients with SCC or patients with AC and ASC with respect to MSLN expression. Kaplan–Meier RFS and OS analysis with respect to MSLN expression. (A) RFS and (B) OS in SCC patients with high MSLN expression versus those in patients with low MSLN expression. (C) RFS and (D) OS in AC and ASC patients with high MSLN expression versus those in patients with low MSLN expression. AC: adenocarcinoma; ASC: adenosquamous carcinoma; MSLN: mesothelin; OS: overall survival; RFS: relapse-free survival; SCC: squamous cell carcinoma. [file 12885_2022_10277_MOESM1_ESM.pptx]

## Slide 1
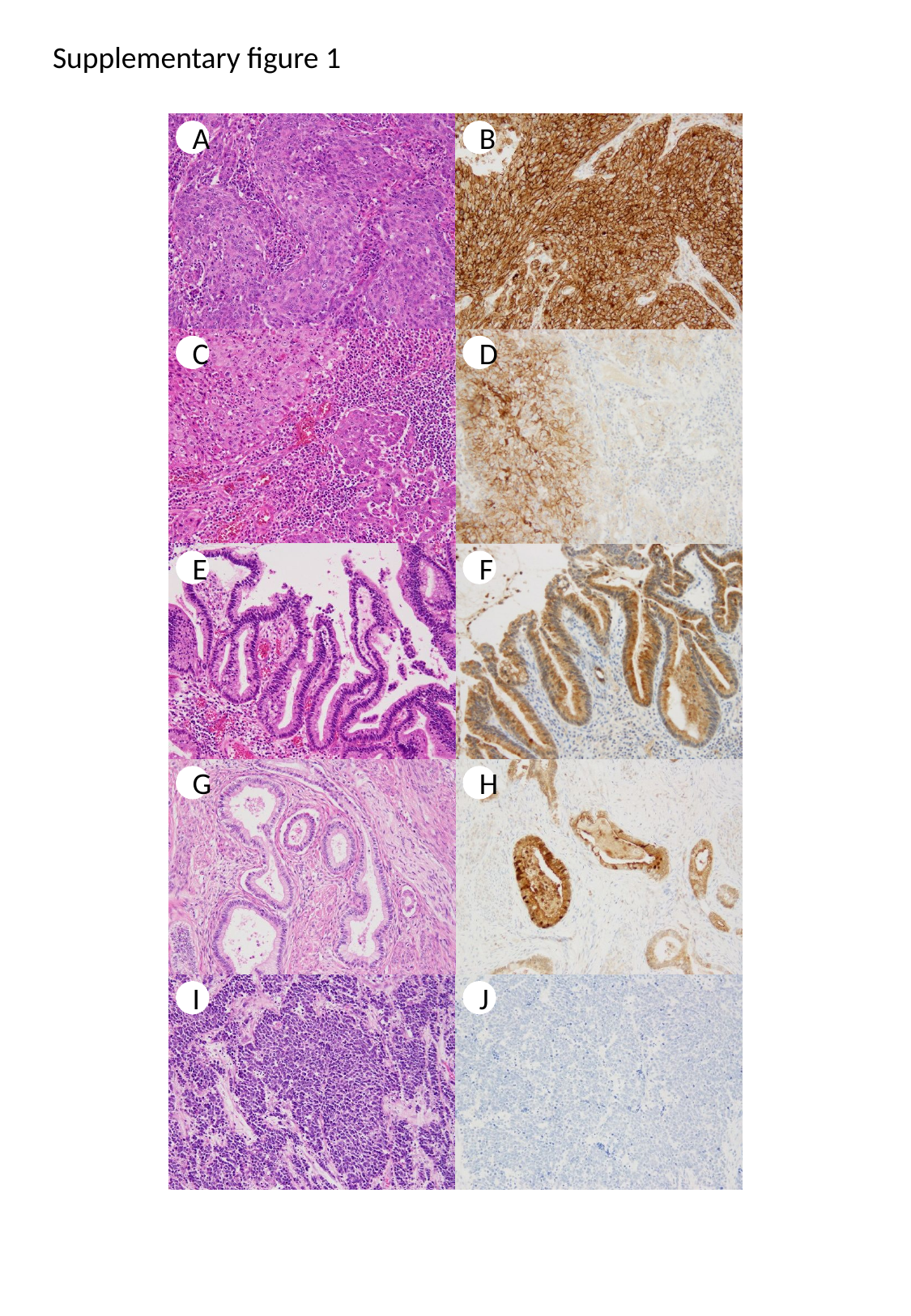

Supplementary figure 1
A
B
C
D
E
F
G
H
I
J

## Slide 2
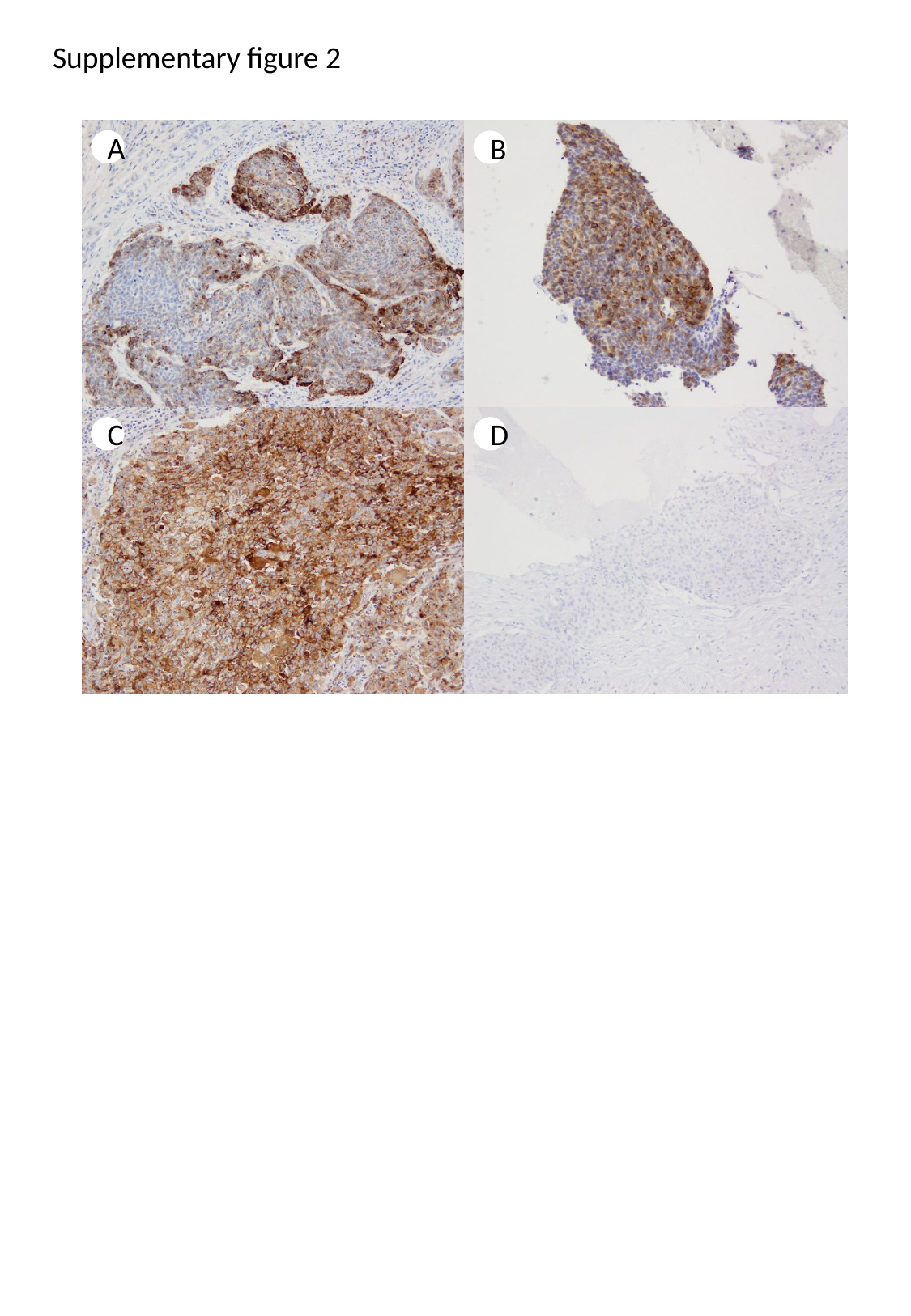

Supplementary figure 2
A
B
C
D

## Slide 3
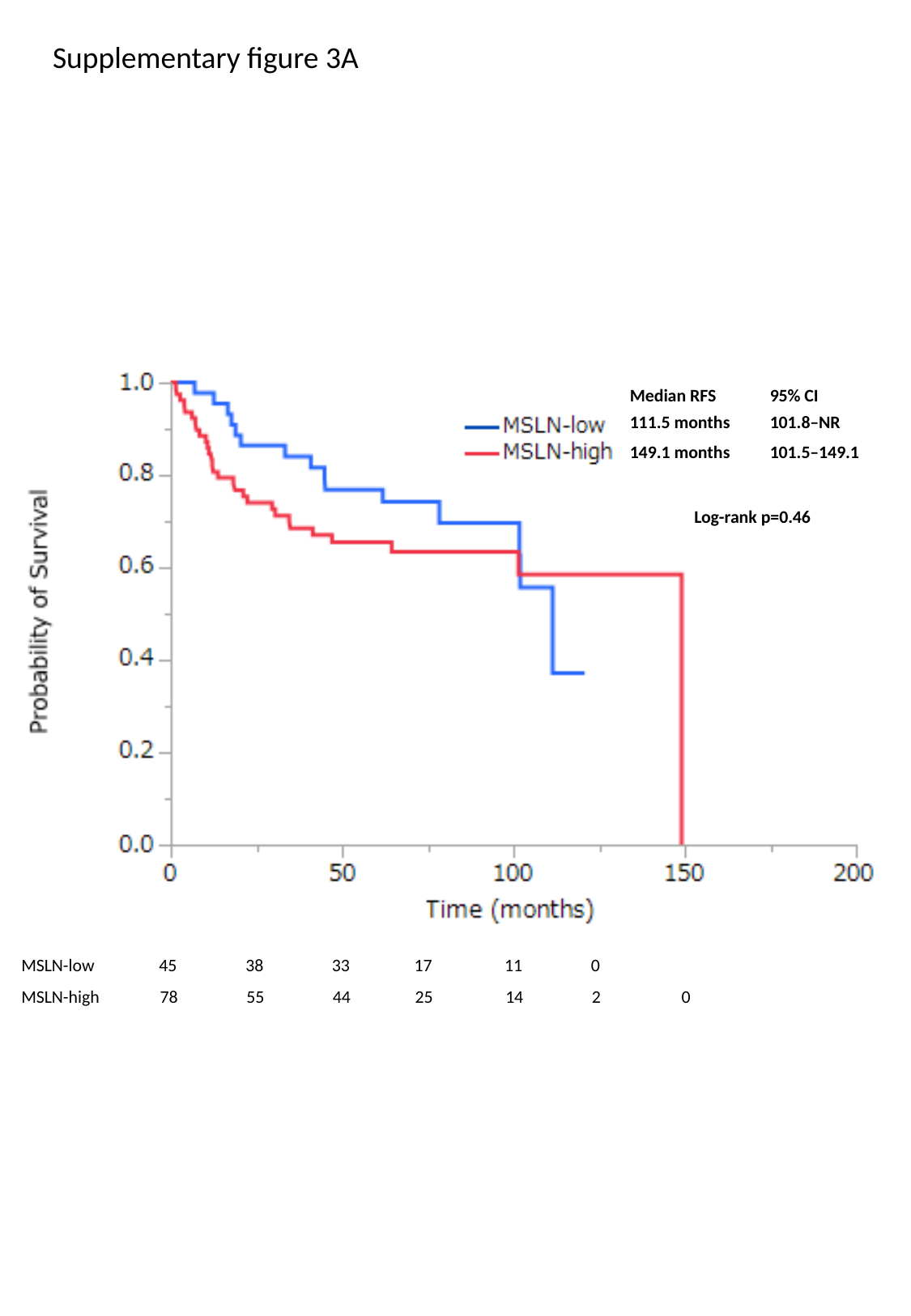

Supplementary figure 3A
| Median RFS | 95% CI |
| --- | --- |
| 111.5 months | 101.8–NR |
| 149.1 months | 101.5–149.1 |
Log-rank p=0.46
MSLN-low 45 38 33 17 11 0
MSLN-high 78 55 44 25 14 2 0

## Slide 4
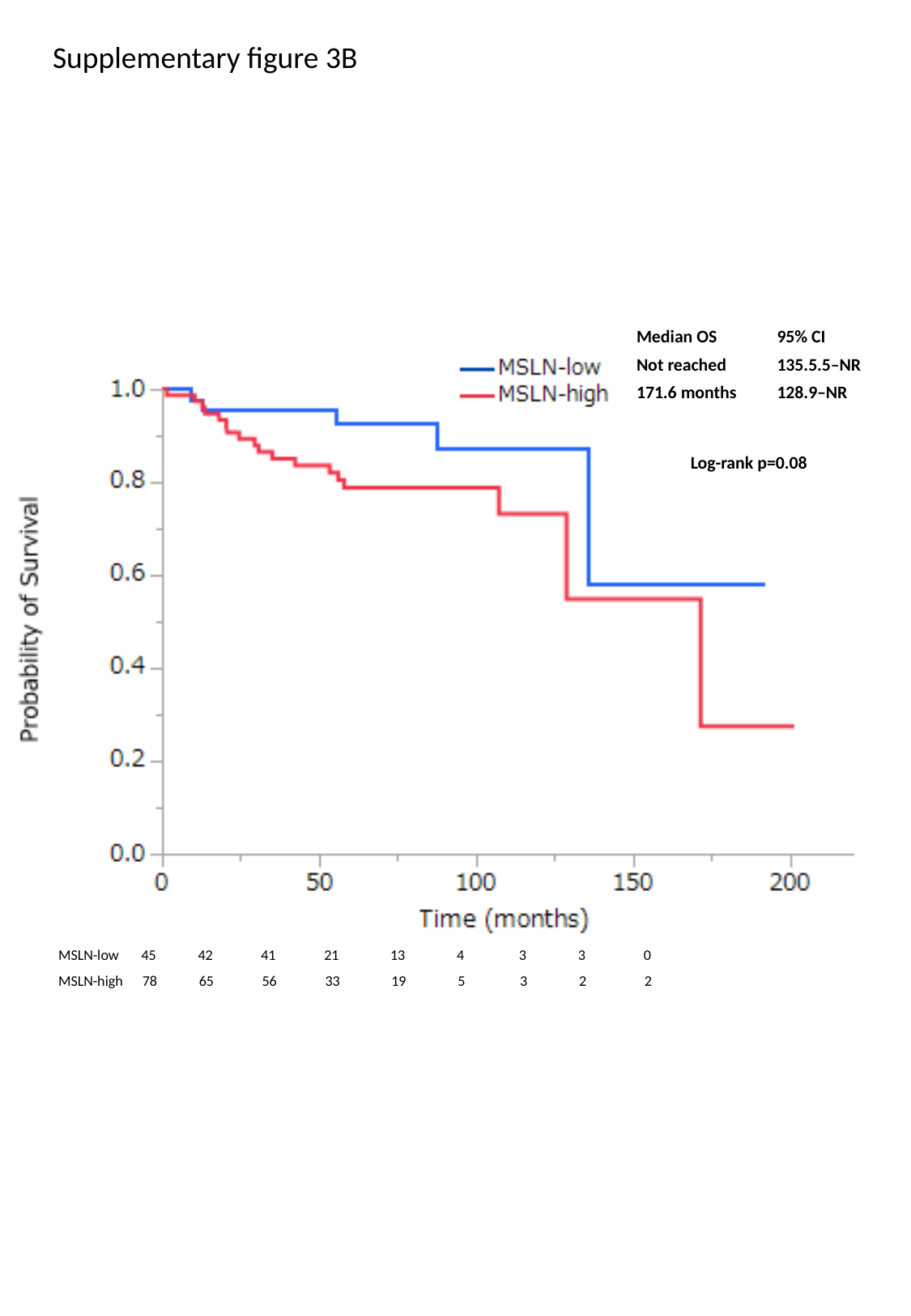

Supplementary figure 3B
| Median OS | 95% CI |
| --- | --- |
| Not reached | 135.5.5–NR |
| 171.6 months | 128.9–NR |
Log-rank p=0.08
MSLN-low 45 42 41 21 13 4 3 3 0
MSLN-high 78 65 56 33 19 5 3 2 2

## Slide 5
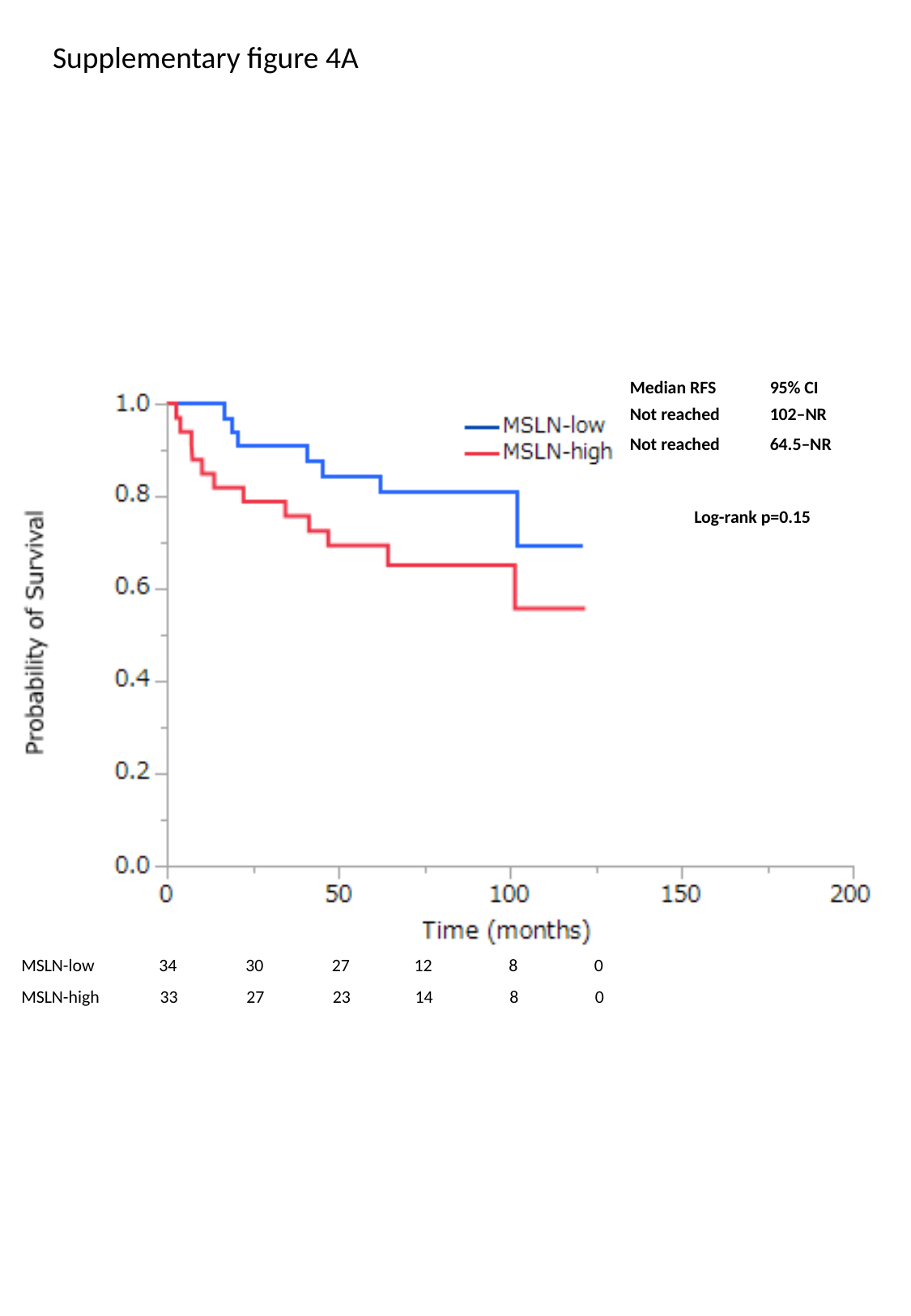

Supplementary figure 4A
| Median RFS | 95% CI |
| --- | --- |
| Not reached | 102–NR |
| Not reached | 64.5–NR |
Log-rank p=0.15
MSLN-low 34 30 27 12 8 0
MSLN-high 33 27 23 14 8 0

## Slide 6
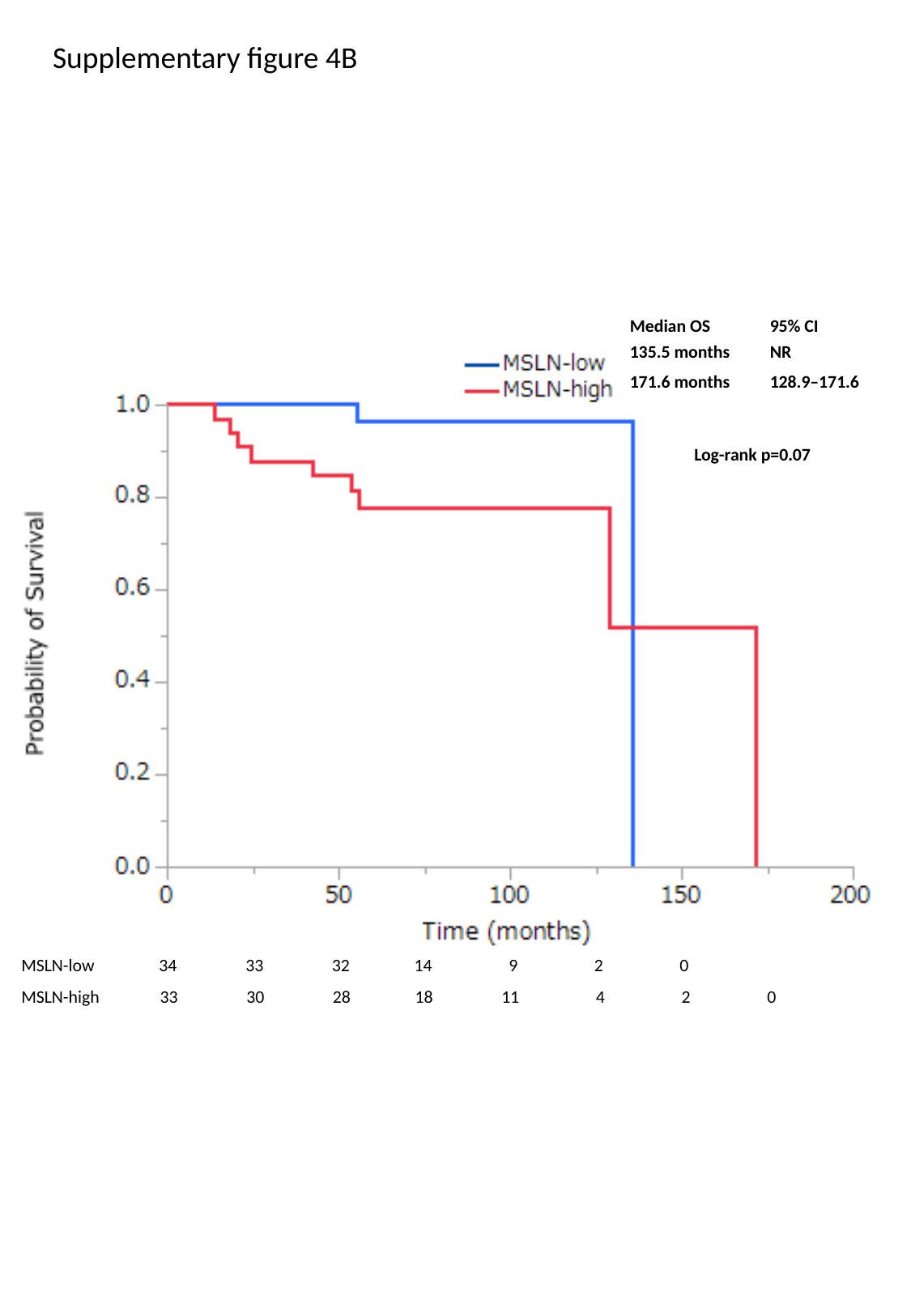

Supplementary figure 4B
| Median OS | 95% CI |
| --- | --- |
| 135.5 months | NR |
| 171.6 months | 128.9–171.6 |
Log-rank p=0.07
MSLN-low 34 33 32 14 9 2 0
MSLN-high 33 30 28 18 11 4 2 0

## Slide 7
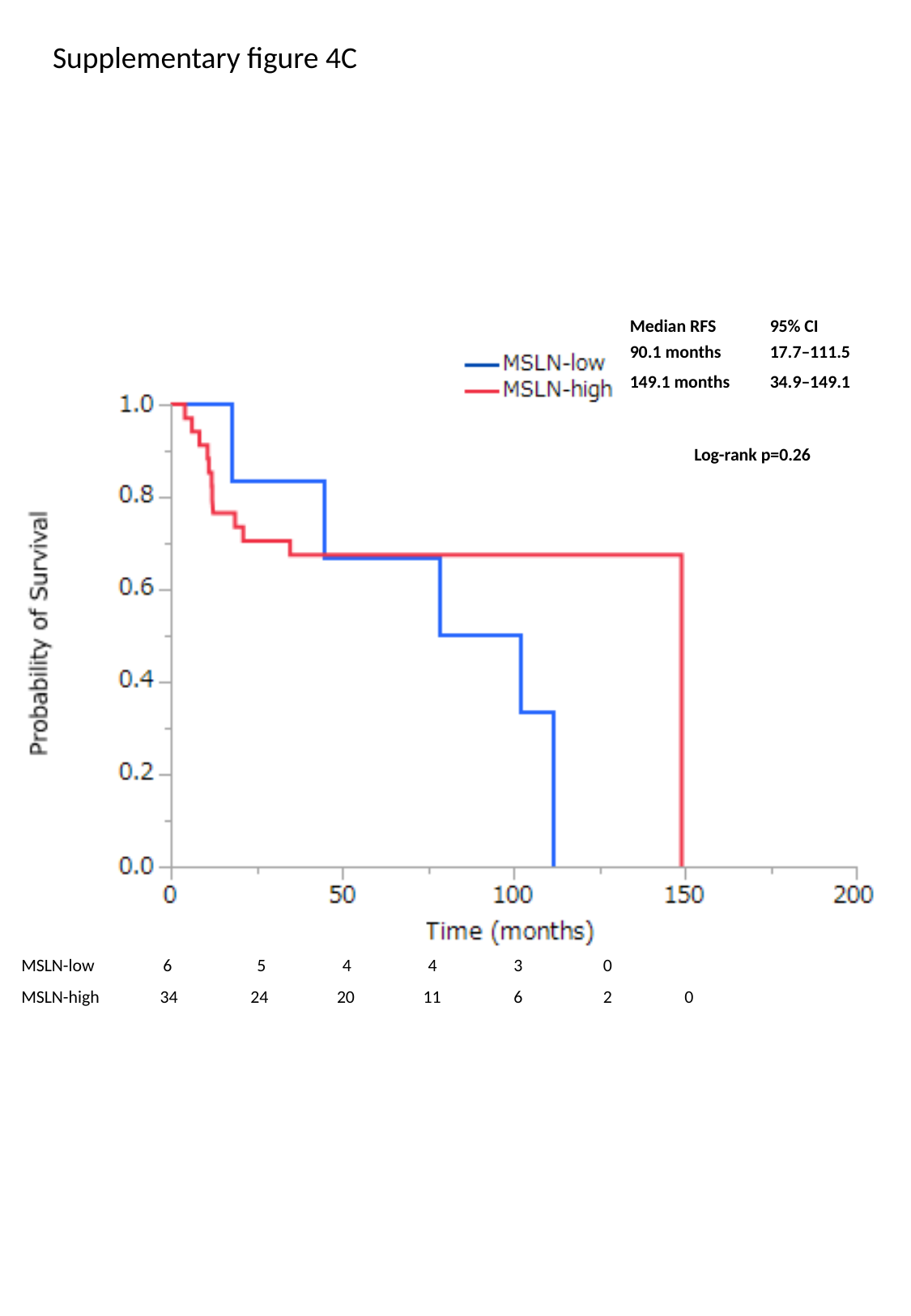

Supplementary figure 4C
| Median RFS | 95% CI |
| --- | --- |
| 90.1 months | 17.7–111.5 |
| 149.1 months | 34.9–149.1 |
Log-rank p=0.26
MSLN-low 6 5 4 4 3 0
MSLN-high 34 24 20 11 6 2 0

## Slide 8
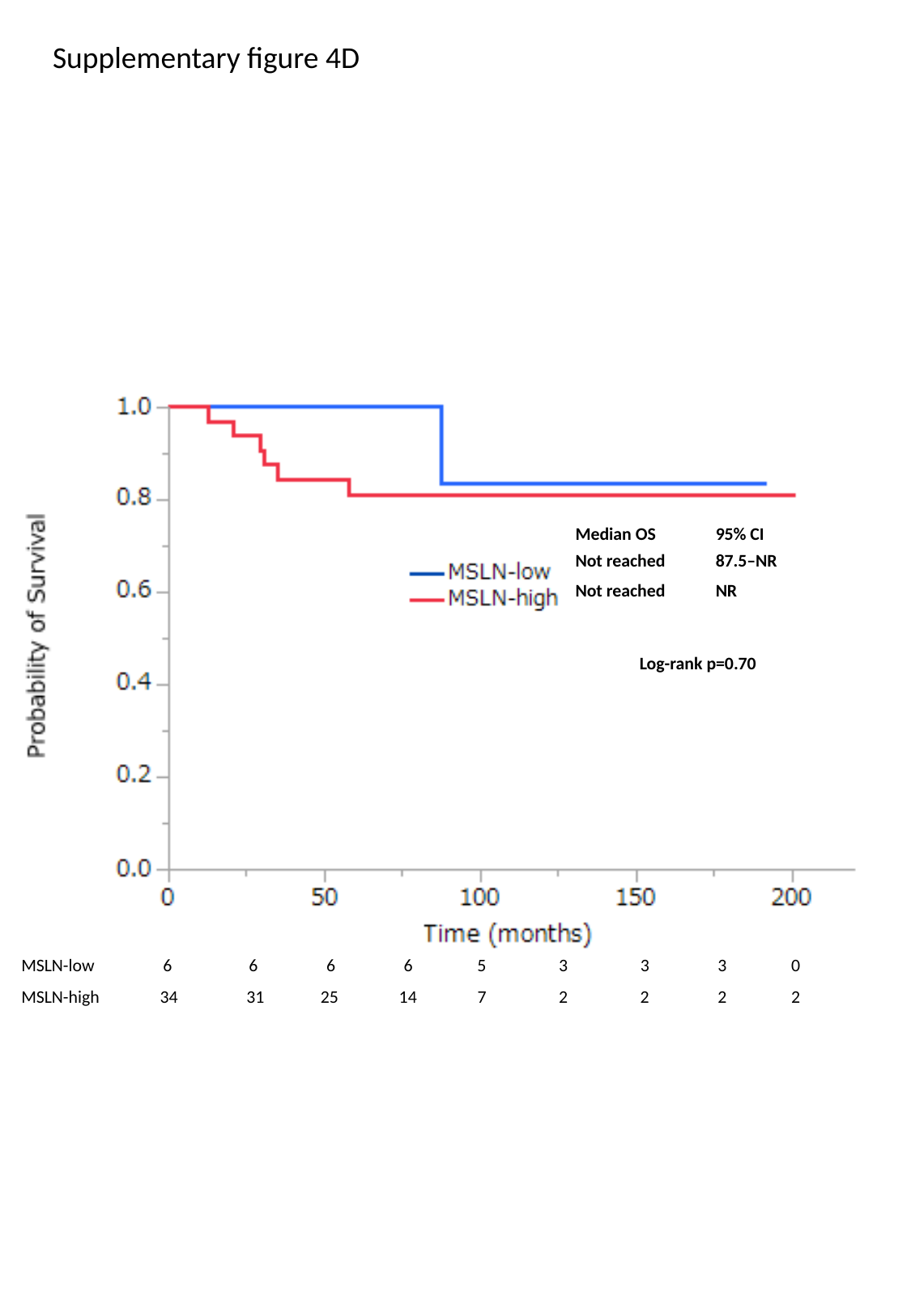

Supplementary figure 4D
| Median OS | 95% CI |
| --- | --- |
| Not reached | 87.5–NR |
| Not reached | NR |
Log-rank p=0.70
MSLN-low 6 6 6 6 5 3 3 3 0
MSLN-high 34 31 25 14 7 2 2 2 2
