# Supplementary material for: High mesothelin expression is correlated with non-squamous cell histology and poor survival in cervical cancer: a retrospective study
Source: BMC Cancer. 2022 Nov 24;22:1215. doi: 10.1186/s12885-022-10277-0 (PMC9701073; doi:10.1186/s12885-022-10277-0)
Supplement: Supplementary file 2 — Additional file 2: Supplementary Table 1A. Multivariate analyses of relapse-free survival in 107 patients with common histological types. Supplementary Table 1B. Multivariate analyses of overall survival in 107 patients with common histological types. [file 12885_2022_10277_MOESM2_ESM.docx]

**Supplementary Tables**

**Additional file 2:**

**Supplementary Table 1A. Multivariate analyses of relapse-free survival in 107 patients with common histological types**

| Total (n = 107) | Univariate | | Multivariate | |
| --- | --- | --- | --- | --- |
|  | Hazard ratio (95% CI) | *p* | Hazard ratio (95% CI) | *p* |
| MSLN (High vs. Low) | 1.34 (0.68–2.79) | 0.41 | 0.98 (0.47–2.15) | 0.96 |
| Histology (SCC vs. AC/ASC) | 0.65 (0.34–1.29) | 0.21 | 0.68 (0.33–1.42) | 0.3 |
| FIGO Stage (I vs. II) | 1.15 (0.51–3.08) | 0.75 | 1.34 (0.56–3.73) | 0.53 |
| Lymph node meta (Positive vs. Negative) | 3.21 (1.64–6.46) | **< 0.001** | 3.27 (1.31–8.45) | **0.011** |
| Postoperative RT (Yes vs. No) | 2.07 (1.05–4.13) | **0.035** | 1.01 (0.41–2.6) | 0.98 |
| Postoperative CRT (No vs. Yes) | 1.19 (0.42–4.97) | 0.77 | 0.75 (0.23–3.35) | 0.67 |
| Age (≥ 45 years vs. < 45 years) | 1.41 (0.72–2.83) | 0.32 | 1.20 (0.6–2.47) | 0.6 |

**Supplementary Table 1B. Multivariate analyses of overall survival in 107 patients with common histological types**

| Total (n = 107) | Univariate | | Multivariate | |
| --- | --- | --- | --- | --- |
|  | Hazard ratio (95% CI) | *p* | Hazard ratio (95% CI) | *p* |
| MSLN (High vs. Low) | 3.53 (1.16–15.3) | **0.03** | 3.70 (1.09–17.6) | **0.034** |
| Histology (SCC vs. AC/ASC) | 1.05 (0.41–2.92) | 0.92 | 1.21 (0.43–3.58) | 0.72 |
| FIGO Stage (I vs. II) | 1.09 (0.35–4.71) | 0.90 | 1.70 (0.49–8.32) | 0.43 |
| Lymph node meta (Positive vs. Negative) | 6.36 (2.24–22.6) | **< 0.001** | 3.46 (0.91–16.3) | 0.07 |
| Postoperative RT (Yes vs. No) | 5.58 (1.96–19.8) | **< 0.001** | 2.36 (0.57–11.6) | 0.25 |
| Postoperative CRT (No vs. Yes) | 1.43 (0.39–9.31) | 0.63 | 1.83 (0.29–17.4) | 0.54 |
| Age (≥ 45 years vs. < 45 years) | 1.07 (0.38–3.04) | 0.90 | 1.17 (0.41–3.42) | 0.77 |

AC: adenocarcinoma; ASC: adenosquamous carcinoma; CRT: chemoradiotherapy; CI: confidence interval; FIGO: International Federation of Gynecology and Obstetrics; MSLN: mesothelin; RT: radiotherapy; SCC: squamous cell carcinoma
